# Social skills group training in children with autism spectrum disorder: a randomized controlled trial

**DOI:** 10.1007/s00787-018-1205-1

**Published:** 2018-07-21

**Authors:** Vera Dekker, Maaike H. Nauta, Marieke E. Timmerman, Erik J. Mulder, Lianne van der Veen-Mulders, Barbara J. van den Hoofdakker, Sjoukje van Warners, Leonieke J. J. Vet, Pieter J. Hoekstra, Annelies de Bildt

**Affiliations:** 1 Department of Psychiatry, University of Groningen, University Medical Center Groningen, Groningen, The Netherlands; 2 Accare, University Center for Child and Adolescent Psychiatry, PO Box 660, 9700 AR Groningen, The Netherlands; 3 Department of Clinical Psychology and Experimental Psychopathology, University of Groningen, Groningen, The Netherlands; 4 Department of Psychometrics and Statistics, University of Groningen, Groningen, The Netherlands; 5 GGZ Drenthe, Center for Intellectual Disabilities and Psychiatry, Assen, The Netherlands

**Keywords:** Social skills training, Effectiveness, Autism spectrum disorder, Randomized controlled trial

## Abstract

In 122 high-functioning children with autism spectrum disorder (ASD; 9–13 years; 19 girls), we investigated the effectiveness of a 15-session social skills group training (SST) with and without parent and teacher involvement (PTI) in a randomized controlled trial with three conditions: SST (*n *= 47), SST–PTI (*n *= 51), and care-as-usual (CAU, *n *= 24). Hierarchical linear modeling was used for immediate and 6-month follow-up analyses. Measures were administered before randomization (blind), post-treatment and at follow-up (not blind). Trial registration: Dutch Trial Register; http://www.trialregister.nl; NTR2405. At post-treatment, children in both SSTs had improved significantly more than CAU on the primary outcome, Vineland Socialization (SST: Cohen’s *d* = 0.39; 95% CI − 2.23 to 3.11 and SST–PTI: *d* = 0.43; 95% CI − 2.19 to 3.15) and on the secondary outcome parent-SSRS “Cooperation” (SST: *d *= 0.43; 95% CI − 0.23 to 1.15 and SST–PTI: *d *= 0.45; 95% CI − 0.21 to 1.17), with no difference between post-treatment and follow-up. Additionally, children in SST–PTI improved significantly more on the teacher-SSRS than in CAU [“Cooperation” *d *=0.42 (95% CI − 0.33 to 1.13); “Assertion” *d *=0.34 (95% CI − 0.39 to 1.11); “Self-Control” *d *=0.61 (95% CI − 0.08 to 1.34)] and in SST [“Cooperation” *d *=0.34 (95% CI − 0.37 to 1.05); “Self-Control” *d *=0.59 (95% CI − 0.13 to 1.32)]. The current study corroborates earlier findings in smaller samples and wider age ranges, with small but statistically significant effects of SST for high-functioning pre-adolescent children with ASD. Parental and teacher involvement intensified treatment, yet did not yield an additional effect relative to SST for children only, as reported by parents. 6 months after training, no further improvement or decline was found.

**Electronic supplementary material:**

The online version of this article (10.1007/s00787-018-1205-1) contains supplementary material, which is available to authorized users.

## Introduction

To improve social-communicative skills in children with autism spectrum disorders (ASD), group-based Social Skills Trainings (SSTs) are widely provided in clinical practice. The short-term effectiveness of SST has been demonstrated in two recent meta-analyses [1, 2]. Effect sizes varied for different sources of treatment evaluation. Parents and external observers generally reported small effects of training, teachers reported no effect, and children and adolescents reported large effects, all compared to no-treatment or waiting-list conditions. The latter effects mainly reflect improvement in social knowledge rather than actual social behavior. Besides informants, the exact instruments used seem to affect the effect, with moderate to large parent-reported effects in the meta-analysis [2] on the Social Skills Rating System (SSRS [3]), and Social Responsiveness Scale (SRS [4]).

One of the ultimate aims of SST in ASD is to improve social skills beyond the duration of the training. Since longer term follow-up data are often lacking, the authors of recent meta-analyses could not draw conclusions regarding the long-term effects [1, 2]. For studies with 3 months follow-up assessments for treated and non-treated groups, the outcomes varied: the immediate effect of group-based SST reported by Soorya et al. [5] did not sustain at 3 months’ follow-up, whereas Freitag et al. [6] and Deckers et al. [7] reported a significant effect at 3 months’ follow-up. Choque Olsson et al. [8] did not find an effect of training on children, and the effect found for adolescents was shown immediately post treatment only, and not at 3 months follow-up.

Another important aim of SST is to improve social skills in situations beyond the training situation, i.e., in daily life. Research into SSTs could not draw conclusions about how to reach generalization of skills to real life [9]. One way to improve the generalization of children’s social skills may be involving parents and/or teachers in the SST intervention, reasoning that they can directly support children to put their training into practice in daily life. Wolstencroft et al. [2] showed that all children in an SST improved, independent from parental involvement, yet with a large effect size in the group with parental involvement and moderate in the group without. However, due to other differences between the studies (e.g., participant characteristics, measures used), direct comparisons are complicated.

The current study [Efficacy of Social skills Training In Autism (ESTIA)] is a randomized controlled trial (RCT) into the effectiveness of a manualized group SST with and without parental and teacher involvement for high-functioning children with ASD in the last two and a half years of primary education, in a large and well-characterized sample of 122 children, with a 6-month follow-up in all conditions. We aimed to investigate two main questions: (1) what is the immediate and long-term effect of group-based SST for high-functioning children with ASD compared to no training (care-as-usual, CAU) based on social skills applied in school and in home-based daily life situations? and (2) what is the additional immediate and long-term effect of parental and teacher involvement on generalization of social skills in daily life of children with ASD compared to group-based SST for the children only?

We hypothesized that (1) children in an SST would improve more on measures of social skills compared to children without an SST; (2) children with SST and additional parent and teacher involvement (SST–PTI) would improve more on measures of social skills, compared to children without this support (SST); (3) children with additional support (SST–PTI) would better maintain these skills after training, compared to children without such support (SST).

## Methods

### Design

**Fig. 1:**
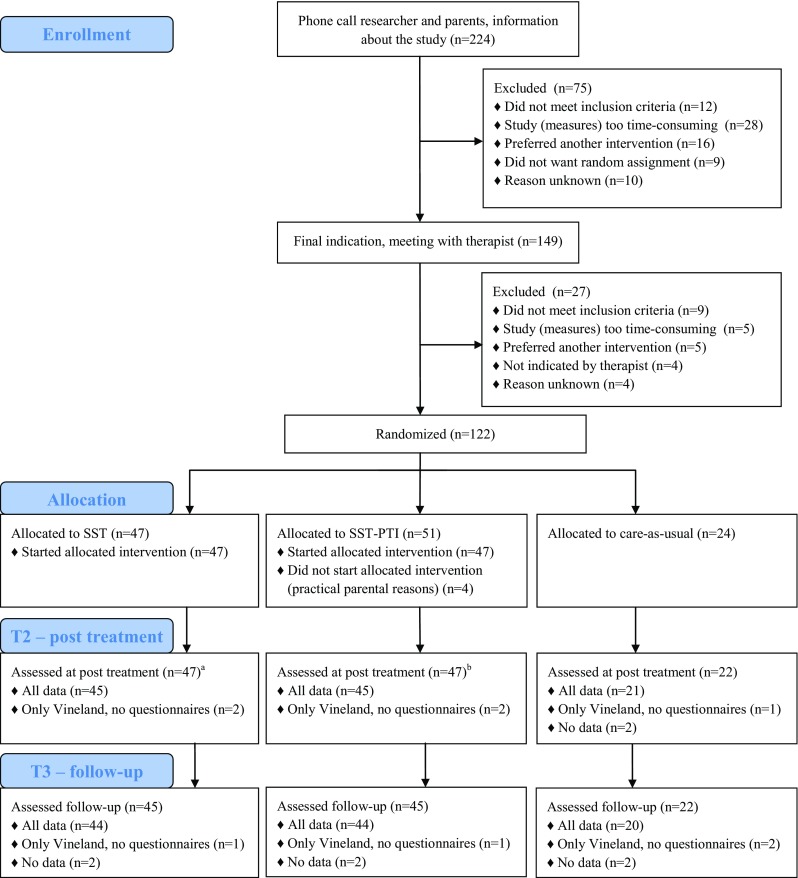
CONSORT diagram of study recruitment, treatment allocation, and assessment. ^a^Two children participated in less than half of the child sessions; ^b^two children participated in less than half of the child sessions, two parents participated in less than half of the parent sessions (one from the same family as one of the children who participated in less than half of the child sessions), one teacher did not participate

The RCT had three conditions: group SST only, group SST–PTI, and care-as-usual without SST. We collected measures at three time points: pre-treatment (T1), immediately post treatment or after the same amount of time in CAU (T2), and follow-up 6 months post treatment or after the same amount of time in CAU (T3). We could only collect teacher information at T1 and T2, due to change of class (and teacher) with changing school year. The first measurement took place before randomization, at later measurements informants or interviewers were not blind to condition. Before participation, parents, teachers, and children (if aged 12) signed an informed consent. The study followed the CONSORT guidelines for RCTs [10] (see Supplementary Table 1 for CONSORT checklist). The Institutional Review Board of the University Medical Center Groningen had approved the study. The study was registered in the Dutch Trial Register (NTR2405; http://www.trialregister.nl). Figure 1 shows the flowchart of study recruitment, treatment allocation, and assessment. For a detailed description, we refer to the research protocol [11].

### Participants

Participants were 122 pre-adolescent high-functioning children with ASD from one of four outpatient mental health care clinics in the northern Netherlands (103 boys, 19 girls). The inclusion criteria were (1) clinical DSM-IV-TR ASD diagnosis [Autistic disorder, Asperger’s disorder, or Pervasive Developmental Disorder-Not Otherwise Specified (PDD-NOS)], based on thorough diagnostic procedures (developmental history, current problems, child observation, and information from school) in expert teams including at least a child psychologist and a child psychiatrist; (2) the child’s clinician indicated SST as first appropriate treatment; (3) parents and child were motivated for SST, as established during a meeting with the clinician, parents and child; (4) preferably IQ ≥ 80. Children with IQs slightly below 80 were included when therapists established they could follow an SST; (5) being in the last two and half years of primary education; (6) no physical condition affecting participation; and (7) the child could travel to the child mental health center for training. Note that three of the criteria were slightly different from the original design registered in the trial register, to more closely approximate the regular decisions in clinical practice. The three original inclusion criteria were (1) ASD diagnosis was either supported by an Autism classification on the Autism Diagnostic Interview-Revised (ADI-R) or maximally two points below the cut off for Autism but with an ASD classification on the ADOS, (2) IQ ≥ 80, and (3) being in the last 2 years of primary education.

Seventeen participants had a DSM-IV-TR diagnosis of autistic disorder (14%), 25 of Asperger’s disorder (20%), and 80 of PDD-NOS (66%). The conditions did not differ on ASD diagnosis (Pearson *χ*^2^ 0.45; *p* = 0.978). Of all children, 32% had one comorbid diagnosis, 4.1% had two comorbid diagnoses (24 Attention Deficit Hyperactivity Disorder, 19.7% of all participants; 8 Tic disorder, 6.5%; four Anxiety Disorder, 3.2%; four Oppositional Defiant Disorder, 3.2%; four other, 3.2%). The conditions did not differ on comorbid secondary diagnoses (Pearson *χ*^2^ 0.39.62; *p* = 0.166) or tertiary diagnoses (Pearson *χ*^2^ 12.61; *p* = 0.246). Mean age at start was 11 years (SD = 0.75; range 9.5–13.0), mean total IQ was 101.5 (SD = 15.3; range 72–135). Male sex proportion was similar over the conditions (Pearson *χ*^2^ 2.63; *p* = 0.268). The conditions did not differ on psychotropic medication use between start and post-treatment. Between post-treatment and follow-up, more children in the CAU condition (22.7%) used anti-psychotic medication compared to SST (6.7%) and SST–PTI (4.4%; Pearson *χ*^2^ 6.55; *p* = 0.038). Most children had at least one parent of Dutch descent (*n* = 121, including 88 with two Dutch parents). Table 1 presents the participant characteristics at baseline.

**Table 1:** Baseline participant characteristics (*N* = 122), including observed sample mean, standard deviation, range and sample size, per condition and per outcome measure

	SST (*N* = 47; 87% male)		SST–PTI (*N* = 51; 78% male)		CAU (*N* = 24; 92% male)		Statistics
	Mean (SD)	Range	Mean (SD)	Range	Mean (SD)	Range	ANOVA *p* value
Age (years)	10.9 (0.7)	9.9–12.6	10.9 (0.8)	9.6–12.7	11.2 (0.9)	9.8–13.0	0.12
ADOS							
Social effect	8.7 (4.5)	2–20	7.9 (3.8)	0–20	8.6 (3.3)	3–15	0.58
Restricted and repetitive behavior	1.0 (0.9)	0–30	1.3 (1.1)	0–50	1.1 (1.2)	0–40	0.38
Calibrated Severity Score	5.6 (2.4)	1–10	5.4 (2.3)	1–10	5.7 (2.1)	2–90	0.85
ADI-R							
Social interaction	15.0 (6.1)	4–27	13.7 (5.6)	3–26	13.7 (4.5)	2–22	0.46
Communication	12.5 (4.9)	2–23	11.3 (4.4)	3–21	12.2 (4.6)	0–19	0.44
Restricted and repetitive behavior	3.0 (2.1)	0–8	3.4 (2.1)	0–10	3.0 (2.2)	0–8	0.70
Vineland							
Communication	111.4 (8.0)	95–126	112.1 (8.6)	88–129	115.3 (8.1)	94–128	0.19
Daily living skills	120.2 (14.7)	70–145	121.2 (13.7)	93–149	125.6 (13.1)	98–150	0.29
Socialization	79.5 (12.9)	53–107	82.4 (16.1)	26–118	86.8 (14.8)	52–115	0.14
ESTIA-TS SSRS-P							
Training-specific social skills	73.0 (15.4)	42–106	68.6 (12.8)	47–102	74.4 (12.5)	53–103	0.15
Cooperation	7.5 (4.1)	1–17	8.2 (3.2)	2–13	7.8 (4.2)	2–16	0.67
Assertion	9.8 (3.0)	4–19	10.6 (3.1)	3–19	10.3 (2.6)	4–15	0.40
Self-control	7.2 (3.5)	0–16	7.7 (3.3)	1–16	7.5 (3.5)	3–13	0.70
Responsibility	9.7 (3.6)	0–18	10.9 (3.2)	3–17	10.7 (2.4)	7–17	0.20
SSRS-T							
Cooperation	13.5 (4.0)	4–20	13.0 (4.7)	2–20	12.4 (4.5)	7–20	0.59
Assertion	9.2 (4.1)	0–16	8.2 (4.0)	1–17	8.5 (4.2)	0–17	0.52
Self-control	10.5 (4.1)	3–20	9.0 (4.3)	2–18	9.0 (3.7)	4–18	0.15
IQ	102.5 (14.8)	72–135	98.7 (16.4)	73–132	105.6 (13.1)	73–126	0.17

*ADOS* autism diagnostic observation schedule, *CAU* care-as-usual, *ESTIA-TS* efficacy of social skills training in autism—training specific, *SSRS-P* social skills rating scale-parents, *SSRS-T* social skills rating scale-teacher, *SST* social skills training, *SST–PTI* social skills training—parent and teacher involvement, *Vineland* Vineland Adaptive Behavior Scales

### Interventions

SST was manualized, based on behavioral therapeutic principles and the social learning theory (Van Warners, Vet, Van der Veen-Mulders and Van den Hoofdakker, 2010; internal publication). The training had 15 weekly 90 min basic group sessions and three additional 90 min booster group sessions, planned between 2 and 6 months after the 15th session. Each session followed a structure: conversation, homework review, introducing a new topic, practice and role play, new homework, and play time. Children received a workbook with summaries of the trained skills and homework. The goal of the first four sessions was to create a safe environment. Sessions 5 through 15 covered specific topics, e.g., “asking something to someone”, “responding to bullying”. A full overview of the session topics can be found in Supplementary Table 2. Children received training through instruction, directed positive feedback, observation, role play, and homework. The therapists analyzed the behavior of the children, defined individual positive target behaviors and elicited positive behavior. Negative behavior was ignored when possible, while differentially reinforcing alternative or incompatible positive behavior. In the booster sessions, children rehearsed their individual target behaviors. The SST groups consisted of four to six children, led by two therapists, i.e., psychologists with at least a psychology master. The therapists received training in the SST by behavioral therapists before and supervision during SST to increase treatment integrity. We refer to the research protocol [11] for a more comprehensive description of the procedures for therapists.

The SST–PTI condition consisted of the SST, with additional parent and teacher involvement (Van Warners and Vet, 2010; internal publication), aiming to enhance generalization of learned social skills. Parents participated in three group sessions before and five during SST. The first three sessions covered psycho-education and interventions for enhancing desired behaviors. The other sessions were related to the SST sessions and focused on how to support the child in practicing the trained social skills. Parent sessions included instruction, behavioral exercises, role play, and practicing learned skills at home.

Teacher support was provided through teacher–therapist meetings before the SST, to discuss the training and the skills aimed to address. During SST, the teacher had five telephone meetings with the therapist to discuss opportunities to support the child in practicing skills at school.

Participants in the CAU condition did not receive SST, defined as a manualized, child-specific training or program. Parent counseling, not focused on social skills, was allowed, e.g., psycho-education, counseling for family functioning, support in finding the right school or medication control, depending on the need of each participant. The conditions did not differ in the CAU delivered. Sessions with parents, with the child, or with parents and child, telephone contact, medication control, personalized support at home or school, a special program in school, or other help or support were equally present in all conditions, as reported by parents. After follow-up, children from CAU could enroll in SST.

In all conditions, delivery of SST outside the study was monitored.

### Treatment fidelity and adherence

After each session, therapists rated whether they had addressed each component of the session. Therapists adhered to the protocol in 97.6% of the time in the SST basic sessions, 97.5% in the parent sessions, 93.3% in the initial teacher session, and 86.5% in the children’s booster sessions.

### Baseline assessment

The severity of ASD symptomatology was measured with the Autism Diagnostic Interview-Revised (ADI-R [12]) and the Autism Diagnostic Observation Schedule (ADOS) module 3 [13, 14], by trained psychologists who met research requirements for reliability. Cognitive ability was assessed with the Wechsler Intelligence Scale for Children—3rd edition (WISC-III [15]).

### Outcome measures

Our primary outcome, reflecting the main aim of training and evaluating the effectiveness of SST in daily life, was the level of social functioning as measured with the raw total “Socialization” domain scores of the Vineland Adaptive Behavior Scales—Survey version (Vineland [16]). As secondary outcome measure we evaluated the specific social skills trained during SST with the ESTIA training-specific skills (ESTIA-TS; Vet et al., 2010; unpublished questionnaire). This 30-item parent questionnaire evaluated the difficulty for the child of performing the specific skills taught in the training. We also evaluated the frequency of general social skills in home and school situations as reported by parents and teachers with the subscales “Cooperation”, “Assertion”, “Self-control”, and “Responsibility” of the 38-item Social Skills Rating Scale parent version (SSRS-P) and corresponding subscales “Cooperation”, “Assertion”, and “Self-control” of the 30-item teacher version (SSRS-T [3]).

### Randomization

From September 2010 through September 2013, training groups were started (September and February). All children finished the training within one school year. When four to six participants were included in the study in a setting, they were randomized into one of the conditions as a group, in a 2:2:1 ratio (SST:SST–PTI:CAU), after the first assessment. Randomization was done in blocks of five groups per stratum, based on setting, using a computer-generated list of treatment allocations, performed by research assistants, unaware of the randomization algorithm and unable to access the computer-generated list to conceal the sequence of allocation.

We aimed to include 48 children in both SSTs and 24 children in CAU, based on a required power of 0.99 and a significance level of 0.01 on the primary outcome measure Vineland for the time effect (i.e., from T1 to T3) and the differential time effects between conditions, taking into account a supposed drop-out rate of 10%. The presumed effect sizes across time in the comparison between the SST and CAU groups were based on previous research with the Vineland as outcome measure [17]. For the comparison between the SST and the ST-PTI groups, we considered an effect size of 0.60 SD of the mean to be clinically relevant. Due to the variation in defined differences between the compared groups, a larger amount of children was needed for comparing SST and SST–PTI than for comparing SST to CAU. For a detailed description of the sample size calculation, we refer to the research protocol [11]. Due to differences in group size (four to six participants), the treatment conditions differed in size (SST *n* = 47, ten groups, SST–PTI *n* = 51, ten groups).

### Statistical analyses

First, we tested differences in baseline background variables, age, IQ, and the outcome measures using analyses of variance (ANOVAs) with allocated treatment as independent variable. Second, we evaluated the effectiveness of the treatment conditions compared to CAU with hierarchical linear modeling using the intent-to-treat principle, including all available data points. We built separate models for the Vineland and the subscales of the SSRS-P, SSRS-T, and ESTIA-TS. The structure of the model consisted of three levels: the measurement occasions (level 1) were nested in the participants (level 2) which were nested in the treatment group (level 3). To assess the treatment effects across time, we built a fully multivariate model for each subscale of each outcome measure. The model accounted for effects of measurement occasion (T1, T2, and T3), condition (SST, SST–PTI, and CAU), and its interaction, while only keeping fixed effects that we hypothesized to be present, or that proved significant. We hypothesized time effects for the treatment conditions (SST and SST–PTI) and their interaction, and therefore kept those effects. Furthermore, we expected no difference between conditions at T1, because of randomization, and we expected no improvement on the outcome measures for the children in the CAU condition, thus we only preserved those effects when significant. Dummy coding was used to model the effects of measurement occasion and treatment, taking T1 and the CAU condition, respectively, as reference conditions. We tested the statistical significance of the fixed effects using the approximate *t* test. The exception was testing the differences between the two treatment groups; this was done using the deviance test, comparing the models mentioned above with a model including both treatment groups together in a single category, and contrasted to the CAU. Additionally, we calculated effect sizes (ES, Cohen’s *d*) on pre- versus postmeasurement and for post- versus follow-up-measurement, for the significant condition differences. ES was derived from differences between conditions at time points, based on the estimated fully multivariate model. In all tests, the significance level was *α* = 0.05.

## Results

At baseline, the three conditions did not differ significantly on any measure. Therefore, we excluded those effects from the model (see Table 1). In building the models, the level 3 random effects (i.e., referring to the treatment group) explained only part of the variance for the subscales “Cooperation” and “Self-control” of the SSRS-T, not for the other measures. Therefore, we only included level 3 in the hierarchical linear model for these subscales.

**Fig. 2:**
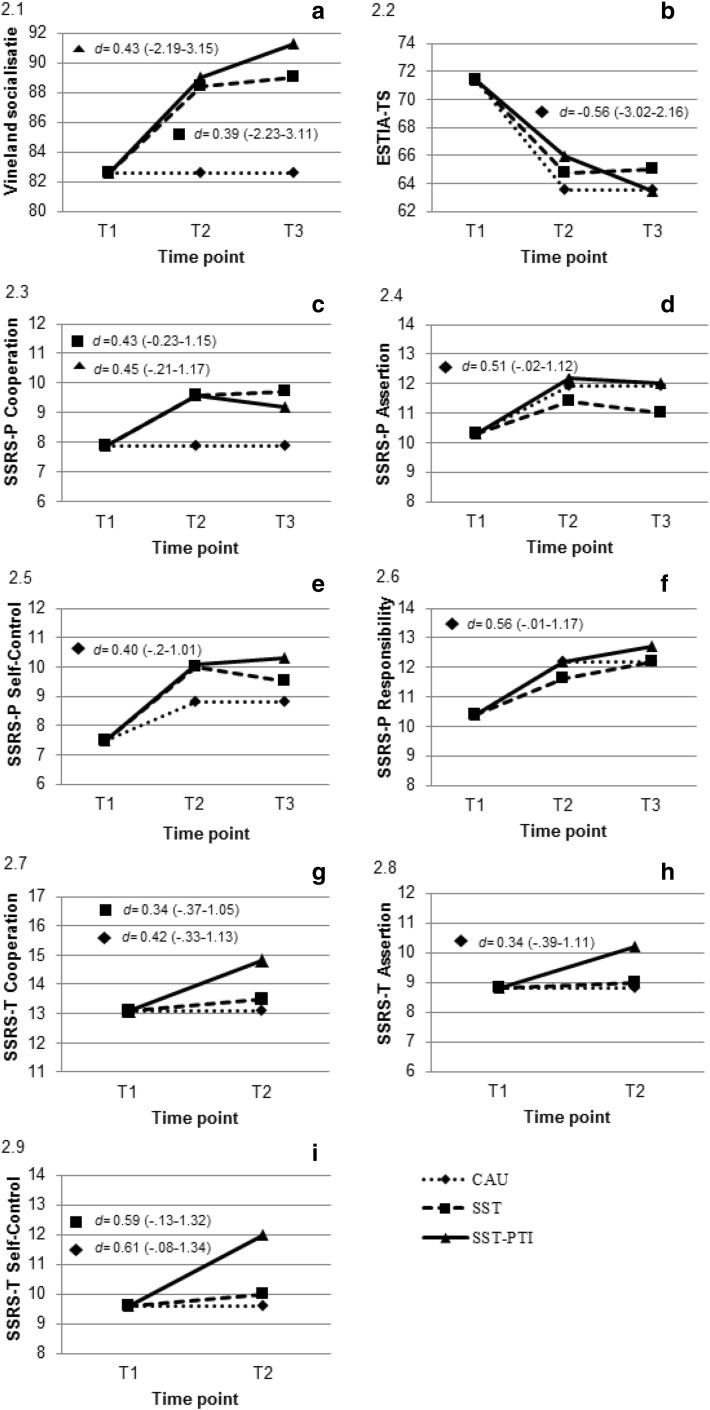
Expected mean per time point and per condition, as based on the fixed part of the multilevel models of the measurements for parents and teachers. Parents: effect sizes in the figures represent significant changes in the specified condition (by symbol), compared to CAU. Significant effect sizes were only present for the comparison between T1 and T2. Teachers: effect sizes in the figures represent significant changes in the social skills training with parent and teacher involvement, compared to CAU (diamond) or compared to SST (square). Each measure is presented in a separate panel. For parent measures: **a** Vineland Socialization; **b** ESTIATS; **c** SSRS-P Cooperation; **d** SSRS-P Assertion; **e** SSRS-P Self-Control; **f** SSRS-P Responsibility. For teacher measures: **g** SSRS-T Cooperation; **h** SSRS-T Assertion; **i** SSRS-T Self-Control

Table 2 presents the estimated coefficients, significance levels, and standard errors of the multilevel models for the parent measures. Table 3 presents these for the teacher measure. The random effects of the models are available upon request from the first author. Figure 2 presents the expected means in the built multi-level model. The actual scores on the instruments in all conditions can be found in the Supplementary Tables S3 and S4.

**Table 2:** Parameter estimates of the multilevel models of the parent measurements (Vineland, SSRS-P, ESTIA-TS)

Fixed effects	Vineland SOC	ESTIA-TS	SSRS-P COO	SSRS-P ASS	SSRS-P SCO	SSRS-P RES
	Estimate (SE)	Estimate (SE)	Estimate (SE)	Estimate (SE)	Estimate (SE)	Estimate (SE)
Intercept (mean score T1 CAU)	82.6 (1.3)^a^	71.4 (1.3)^a^	7.9 (0.3)^a^	10.3 (0.3)^a^	7.5 (0.3)^a^	10.4 (0.3)^a^
Contrast T1–T2 (CAU)	–	− 7.9 (2.1)^a^	–	1.6 (0.5)^b^	1.3 (0.6)^c^	1.8 (0.5)^a^
Contrast T2–T3 (CAU)	–	–	–	–	–	–
SST at T1	–	–	–	–	–	–
SST–PTI at T1	–	–	–	–	–	–
Contrast T1–T2 × SST	5.8 (1.7)^b^	1.2 (2.5)	1.7 (0.4)^a^	− 0.5 (0.7)	1.2 (0.7)	− 0.6 (0.6)
Contrast T1–T2 × SST–PTI	6.4 (1.7)^a^	2.4 (2.5)	1.7 (0.4)^a^	0.3 (0.7)	1.3 (0.7)	0.0 (0.6)
Contrast T2–T3 × SST	0.6 (1.7)	0.3 (1.4)	0.1 (0.3)	− 0.4 (0.4)	− 0.5 (0.4)	0.6 (0.4)
Contrast T2–T3 × SST–PTI	2.3 (1.7)	− 2.5 (1.4)	− 0.4 (0.3)	− 0.2 (0.4)	0.2 (0.4)	0.5 (0.4)

*CAU* care-as-usual, *ESTIA-TS* efficacy of social skills training in autism—training specific, *SSRS-P* social skills rating scale-parents, (*COO* cooperation, *ASS* assertion, sco self-control, *RES* responsibility), *SST* social skills training, *SST–PTI* social skills training—parent and teacher involvement, *Vineland* Vineland Adaptive Behavior Scales (*SOC* socialization),—means that this effect was not included in the model, because it was not hypothesized and appeared nonsignificant when including the effect

^a^*p* < 0.001

^b^*p* < 0.01

^c^*p* < 0.05

**Table 3:** Parameter estimates of the multilevel models of the teacher measurements (SSRS-T)

Fixed effects	SSRS-T COO	SSRS-T ASS	SSRS-T SCO
	Estimate (SE)	Estimate (SE)	Estimate (SE)
Intercept (mean score T1 CAU)	13.1 (0.5)^a^	8.8 (0.4)^a^	9.6 (0.5)^a^
Contrast T1–T2 (CAU)	–	–	–
SST at T1	–	–	–
SST–PTI at T1	–	–	–
Contrast T1–T2 × SST	0.4 (0.5)	0.2 (0.4)	0.4 (0.5)
Contrast T1–T2 × SST–PTI	1.7 (0.5)^a,d^	1.4 (0.4)^b^	2.4 (0.5)^a,d^

*CAU* care-as-usual, *SSRS-T* Social Skills Rating Scale-Teachers (*COO* cooperation, *ASS* assertion, *SCO* self-control), *SST* social skills training, *SST–PTI* social skills training—parent and teacher involvement,—means that this effect was not included in the model, because it was not hypothesized and appeared nonsignificant when including the effect

^a^*p* < 0.001

^b^*p* < 0.01

^c^*p* < 0.05

^d^also a significant difference with SST condition

### Primary outcome

#### Vineland socialization

Children in the SST and the SST–PTI conditions improved significantly more on Vineland Socialization than children in CAU (who did not significantly improve) from T1 to T2 (ES SST: Cohen’s *d* = 0.39; 95% CI − 2.23 to 3.11 and ES SST–PTI: Cohen’s *d* = 0.43; 95% CI − 2.19 to 3.15). SST and SST–PTI did not differ significantly from each other. All conditions showed stable Vineland Socialization scores from T2 to T3. The trajectory of each condition is presented in Fig. 2a.

### Secondary outcomes

#### ESTIA-TS

As shown in Fig. 2b, parents in CAU reported significantly lower difficulty scores for the social skills covered in the training at T2 compared to T1 (ES: Cohen’s *d* = − 0.56; 95% CI − 3.02 to 2.16). From T2 to T3, no significant difference existed for CAU. Parents in both treatment conditions reported a similar pattern from T1 to T2 or T2 to T3.

#### SSRS-P

Figure 2c–f show change as measured with the SSRS. Children in both treatment conditions improved significantly more on “Cooperation” than children in CAU (who did not improve) from T1 to T2 (ES SST: Cohen’s *d *= 0.43; 95% CI − 0.23 to 1.15 and ES SST–PTI: Cohen’s *d *= 0.45; 95% CI − 0.21 to 1.17). They did not differ significantly from each other. From T2 to T3, “Cooperation” scores were stable for all conditions.

On the subscales “Assertion”, “Self-control”, and “Responsibility”, children in CAU improved significantly from T1 to T2. The ES (Cohen’s *d*) were 0.51 (95% CI − 0.02 to 1.12), 0.40 (95% CI − 0.2 to 1.01), and 0.56 (95% CI − 0.01 to 1.17). Children in the CAU condition did not improve significantly from T2 to T3 on these subscales. Both treatment conditions showed similar patterns as CAU.

#### SSRS-T

Children in CAU did not improve significantly on the SSRS-T subscales from T1 to T2 (Fig. 2g–i). Children in the SST condition resembled the CAU condition. Children in the SST–PTI condition improved significantly more between T1 and T2 than CAU, with ES (Cohen’s *d*) 0.42 (95% CI − 0.33 to 1.13) for “Cooperation, 0.34 (95% CI − 0.39 to 1.11) for “Assertion”, and 0.61 (95% CI − 0.08–1.34) for “Self-Control”. On the subscale “Cooperation” and “Self-Control” the children in SST–PTI improved also significantly more than the SST condition, with ES (Cohen’s *d*) 0.34 (95% CI − 0.37 to 1.05) and 0.59 (95% CI − 0.13 to 1.32), respectively.

## Discussion

This study demonstrated that children improved in social functioning in daily life and broad social skills, reported by parents, immediately after group SST. However, no differences existed between the three conditions on the specifically trained social skills and the other SSRS-P subscales. Contrary to our expectations, actively involving parents and teachers in the training did not increase the immediate effect, or the generalization of social skills to situations outside the training or beyond the duration of the training, observed by parents. Six months after training, social skills had not further improved in any group. Note that adding three booster sessions [5, 18] did not contribute to further improvement of social skills either.

The small to medium effect sizes in our study correspond to the findings of Gates et al. [1] in their meta-analysis. They also resemble the results from Deckers et al. [7] in a comparable, Dutch, high-functioning population from a regular outpatient clinic (*n* = 52; effect size 0.34), although measured with a different instrument (the Social Skills Observation; SSO [19]). Compared to the meta-analysis of Wolstencroft et al. [2], we found a smaller effect on the SSRS, and only for the subscale “Cooperation”. Perhaps this difference in effect size is due to the significant improvement of children in the CAU condition in the current study on the subscales “Assertion”, “Self-control”, and “Responsibility”. Although this could reflect natural growth in time, other explanations are also possible. First, parents may have changed in their observation, e.g., the assessment may have included behaviors that parents had not noticed before the first measurement, yet actively looked for after completing the SSRS for the first time. Second, all children in this study, including the children in CAU, wanted to improve social skills and were motivated for training by definition of the inclusion criteria. Parents of children in the CAU condition may thus have tried to improve their child’s social skills in other ways (e.g., reading about social skills/ASD, explaining social situations, stimulating their child to make play dates). Third, many children with PDD-NOS (66%) and the relatively high-functioning character of the sample, may have affected the outcomes.

Our study is one of few that compared long-term outcomes in the treated and CAU conditions [1]. Two earlier studies reported a significant effect after 3 months [6, 7], and no effect was found after 3 months in two other studies [5, 8]. We expected children in the SST–PTI condition to continue improving, based on their parents’ training in how to support them learn and apply social skills. However, this support did not seem to affect further development after training. Practicing may have evanesced after training was over, with its accompanying homework. Moreover, social skills appeal to social insight, and the question is whether that can be trained in a (15-session) SST.

Teachers only reported significant improvement after SST–PTI compared to CAU (all subscales) and SST (“Cooperation” and “Self-control” subscales of the SSRS-T). This finding could indicate the effect of parent and teacher involvement in the training on generalization of learned skills to the school situation. Alternatively, teachers more intensively involved may see improvement for other reasons, e.g., they may have learned how to observe or interpret social behaviors, or they may expect change after their effort in the training, and conform to the expectancy bias in parents [1, 2]. Although in line with the teacher findings of Deckers et al. [7], our finding is in contrast with the meta-analysis of Gates et al. [1], who found no effect of SST as reported by teachers. Since we have no follow-up data of the teachers, we cannot draw conclusions on generalization to the school situation beyond the duration of the training.

### Limitations

Unfortunately, parents and teachers were not blind for condition in the post-treatment measures. A blind observation of the child in a naturalistic situation [20–22], added to parent and teacher report, would have contributed to a more reliable measure of changes in social skills after training, unbiased by either knowledge of treatment condition or actual contributions to training. However, no well-described and valid instruments were available for such observation. Gates et al. [1] found only five studies in their meta-analysis reporting on observer information. Even in this small sample all studies used other instruments, ranging from the ADOS, measuring social communication in a semi-structured situation with one adult, to 5-min observations of mother–child interaction in a structured situation (playing a puzzle), and to peer interactions measured with different instruments.

Additionally, we only used general social skills measures, including social functioning in daily life, and did not focus on ASD-specific social skills. For future studies, we recommend to add ASD-specific social skills measures. We are aware that the Vineland was not developed as an outcome measure for SST. However, using it as our primary measure was an approach to assessing improvements in social functioning in daily life, the ultimate aim of SST. Earlier SST studies including the Vineland did not report strong effects either, i.e., only trending significant changes [17], no differential changes [18], or mixed results on various subscales [23].

### Implications

The current study corroborates earlier findings in smaller samples and wider age ranges, indicating small but statistically significant effects of SST in daily life for high-functioning pre-adolescent children with ASD. Parental and teacher involvement intensified the treatment for therapists, parents and teachers, yet did not yield the expected additional effect relative to SST for children only as reported by parents. More research on who benefits from what form of SST is needed, to enable clinicians to decide who to provide with (what form of) SST.

## Electronic supplementary material

Below is the link to the electronic supplementary material.

Supplementary material 1 (DOC 218 kb)

Supplementary material 2 (DOCX 20 kb)

Supplementary material 3 (DOCX 20 kb)
