# Supplementary material for: Social skills group training in children with autism spectrum disorder: a randomized controlled trial
Source: Eur Child Adolesc Psychiatry. 2018 Jul 21;28(3):415–24. doi: 10.1007/s00787-018-1205-1 (PMC6407743; doi:10.1007/s00787-018-1205-1)
Supplement: Supplementary file 2 — Supplementary material 2 (DOCX 20 kb) [file 787_2018_1205_MOESM2_ESM.docx]

Supplementary Table 2: Topics of the social skills training

| ***Children sessions (in SST and SST-PTI)*** | | |
| --- | --- | --- |
| 1 | Phase 1 (weekly):  Create a safe  environment | Introduction |
| 2 |  | Saying nice things about yourself and to others |
| 3 |  | Feelings (showing how you feel and see how another feels) |
| 4 |  | Personal presentation (posture, eye contact and use of voice) |
| 5 | Phase 2 (weekly):  Practice skills | Asking something to someone |
| 6 |  | Conversation |
| 7 |  | Asking for a play date |
| 8 |  | Asking to participate |
| 9 |  | Discussing with someone |
| 10 |  | Playing a social game |
| 11 |  | Saying no |
| 12 |  | Indicating annoyance |
| 13 |  | Apologizing to someone |
| 14 |  | Responding to bullying |
| 15 |  | Final session, children chose a social activity |
| 16 | Phase 3 (2 weekly-monthly):  Booster sessions | Repeating the above mentioned skills, focusing on individual goals |
| 17 |  | Repeating the above mentioned skills, focusing on individual goals |
| 18 |  | Repeating the above mentioned skills, focusing on individual goals |

| ***Parents sessions (in SST-PTI)*** | | |
| --- | --- | --- |
| 1 | Phase 1 (weekly): Before child sessions | Psycho-education |
| 2 |  | Antecedent interventions |
| 3 |  | Consequent interventions |
| 4 (SST 2) | Phase 2 (2 weekly): During child sessions | Discrimination training |
| 5 (SST 4) |  | Eliciting desired behaviors and creating opportunities |
| 6 (SST 6) |  | Energizing desired social behavior |
| 7 (SST 8) |  | Responding to and redirecting socially awkward behaviour |
| 8 (SST 11) | Phase 3 | Continuation and persevere |

**

| ***Teacher (in SST-PTI)*** |
| --- |
| One meeting with the therapist before the start of the SST for the children en five telephone contacts during the SST (after session 2, 4, 7, 10 and 13). |
