# Supplementary material for: Social skills group training in children with autism spectrum disorder: a randomized controlled trial
Source: Eur Child Adolesc Psychiatry. 2018 Jul 21;28(3):415–24. doi: 10.1007/s00787-018-1205-1 (PMC6407743; doi:10.1007/s00787-018-1205-1)
Supplement: Supplementary file 3 — Supplementary material 3 (DOCX 20 kb) [file 787_2018_1205_MOESM3_ESM.docx]

Supplementary table 3: Per outcome measure and condition, the observed sample mean, standard deviation, range and sample size at T2

|  | | **SST** | |  | **SST-PTI** | |  | **CAU** | |  |
| --- | --- | --- | --- | --- | --- | --- | --- | --- | --- | --- |
|  | | mean (SD) | Range | n | mean (SD) | Range | n | mean (SD) | Range | n |
| **Age** Years | | 11.5 (0.6) | 10.5-12.9 | 47 | 11.4 (0.8) | 10.1-13.2 | 47 | 11.8 (0.9) | 10.3-13.7 | 22 |
| **ESTIA-TS** | Training-specific social skills | 65.8 (16.3) | 34-98 | 46 | 63.3 (13.6) | 38-94 | 45 | 66.8 (14.2) | 44-106 | 22 |
| **SSRS-P** | Cooperation | 9.3 (4.2) | 1-19 | 47 | 9.7 (3.6) | 4-18 | 46 | 8.9 (4.1) | 2-16 | 22 |
|  | Assertion | 11.1 (3.6) | 0-19 | 47 | 12.3 (3.1) | 6-20 | 46 | 11.8 (3.6) | 6-19 | 22 |
|  | Self-Control | 9.9 (3.5) | 2-19 | 47 | 10.2 (2.8) | 3-16 | 46 | 9.4 (4.2) | 0-16 | 22 |
|  | Responsibility | 11.2 (3.7) | 2-19 | 47 | 12.5 (2.9) | 6-18 | 46 | 12 (3.4) | 6-17 | 22 |
| **SSRS-T** | Cooperation | 13.7 (4.1) | 4-20 | 46 | 14.7 (4.4) | 2-20 | 47 | 13.3 (4.0) | 5-20 | 23 |
|  | Assertion | 9.2 (4.1) | 2-19 | 46 | 9.7 (4.3) | 3-20 | 47 | 10.0 (4.1) | 1-20 | 23 |
|  | Self-Control | 10.7 (4.5) | 1-19 | 46 | 11.4 (4.2) | 2-20 | 47 | 9.3(4.0) | 3-18 | 23 |
| **Vineland** | Socialization domain | 86.3 (15.8) | 47-114 | 47 | 88.7 (15.0) | 34-122 | 47 | 88.6 (12.5) | 58-105 | 22 |

Supplementary table 4: Per outcome measure and condition, the observed sample mean, standard deviation, range and sample size at T3

|  | | **SST** | |  | **SST-PTI** | |  | **CAU** | |  |
| --- | --- | --- | --- | --- | --- | --- | --- | --- | --- | --- |
|  | | mean (SD) | Range | n | mean (SD) | Range | n | mean (SD) | Range | n |
| **Age** Years | | 11.9 (0.7) | 10.9-13.6 | 45 | 11.9 (0.8) | 10.5-13.9 | 45 | 12.3 (0.8) | 10.8-14.0 | 22 |
| **ESTIA-TS** | Training-specific social skills | 67.3 (16.8) | 36-101 | 44 | 61.5 (12.7) | 38-85 | 44 | 65.9 (15.1) | 42-96 | 20 |
| **SSRS-P** | Cooperation | 9.3 (3.8) | 2-18 | 44 | 9.3 (3.6) | 3-17 | 44 | 8.5 (4.0) | 2-16 | 20 |
|  | Assertion | 10.6 (3.7) | 4-18 | 44 | 12.3 (3.0) | 6-19 | 44 | 12.3 (3.9) | 5-20 | 20 |
|  | Self-Control | 9.5 (3.8) | 2-19 | 44 | 10.4 (2.9) | 5-17 | 44 | 8.5 (3.7) | 2-13 | 20 |
|  | Responsibility | 11.9 (3.2) | 7-19 | 44 | 13.1 (3.0) | 5-18 | 44 | 13.0 (3.6) | 8-19 | 20 |
| **Vineland** | Socialization domain | 86.8 (16.5) | 49-115 | 45 | 91.5 (15.1) | 41-120 | 45 | 89.8 (16.0) | 61-120 | 22 |
